# Supplementary material for: Synthesis and characterization of geminis and implications of their micellar solution on ninhydrin and metal amino acid complex
Source: R Soc Open Sci. 2020 Jul 29;7(7):200775. doi: 10.1098/rsos.200775 (PMC7428287; doi:10.1098/rsos.200775)
Supplement: Implications of [gemini] on k on [Cu(II)-Trp]+ (0.2 mmol.kg−1) and ninhydrin (6.0 mmol.kg−1) reaction at temperature (353 K) and pH (5.0); and their comparison with k cal. [file rsos200775supp1.docx]

**Supplementary Materials**

**Synthesis and characterization of geminis and implications of their micellar solution on ninhydrin and metal amino acid complex**

**Dileep Kumar^1,2^, Malik Abdul Rub^3,4^** **and Abdullah M. Asiri^3,4^**

^1^Division of Computational Physics, Institute for Computational Science, Ton Duc Thang University, Ho Chi Minh City, Vietnam

^2^Faculty of Applied Sciences, Ton Duc Thang University, Ho Chi Minh City, Vietnam

^3^Chemistry Department, Faculty of Science, King Abdulaziz University, Jeddah-21589, Saudi Arabia

^4^Center of Excellence for Advanced Materials Research, King Abdulaziz University, Jeddah-21589, Saudi Arabia

**Authors for correspondence:**

Dileep Kumar

email: dileepkumar@tdtu.edu.vn

**Table S1.** Implications of [gemini] on *k*_ψ_ on [Cu(II)-Trp]^+^ (0.2 mmol.kg^-1^) and ninhydrin (6.0 mmol.kg^-1^) reaction at temperature (353 K) and pH (5.0); and their comparison with *k*_Ψcal_.

| 10^2^ [gemini]  (mmol.kg^-1^) | 16-6-16 16-5-16 | | |  | 16-4-16 | |
| --- | --- | --- | --- | --- | --- | --- |
|  | 10^5^ *k*_ψ_  (s^-1^) | 10^5^ *k*_ψcal_  (s^-1^) | 10^5^ *k*_ψ_  (s^-1^) | 10^5^ *k*_ψcal_  (s^-1^) | 10^5^ *k*_ψ_  (s^-1^) | 10^5^ *k*_ψcal_  (s^-1^) |
| 0.0 | 2.0 | **-** | 2.0 | **-** | 2.0 | **-** |
| 1.0 | 2.2 | **-** | 2.5 | **-** | 3.1 | **-** |
| 3.0 | 2.4 | **-** | 2.9 | **-** | 3.9 | **-** |
| 5.0 | 2.7 | **-** | 3.3 | **-** | 4.8 | **-** |
| 10.0 | 3.5 | 3.2 | 5.5 | 5.2 | 7.5 | 7.7 |
| 20.0 | 7.0 | 6.8 | 8.8 | 8.7 | 10.5 | 10.5 |
| 30.0 | 9.5 | 9.7 | 10.5 | 10.6 | 12.0 | 12.1 |
| 40.0 | 9.7 | 9.7 | 10.6 | 10.6 | 12.2 | 11.9 |
| 50.0 | 9.9 | 10.1 | 10.7 | 10.9 | 12.4 | 12.5 |
| 60.0 | 10.1 | 10.3 | 10.9 | 11.0 | 12.5 | 12.5 |
| 80.0 | 10.2 | 10.2 | 11.0 | 10.9 | 12.6 | 12.8 |
| 100.0 | 10.3 | 10.1 | 11.1 | 10.8 | 12.8 | 12.6 |
| 250.0 | 10.5 | 10.4 | 11.3 | 11.3 | 13.0 | 13.1 |
| 400.0 | 10.6 | 10.8 | 11.6 | 11.5 | 13.3 | 13.4 |
| 600.0 | 10.8 | 11.0 | 11.9 | 11.8 | 13.9 | 14.0 |
| 1000.0 | 11.6 | - | 13.2 | **-** | 15.2 | - |
| 1500.0 | 12.6 | **-** | 14.5 | **-** | 16.6 | - |
| 2000.0 | 14.0 | **-** | 16.0 | **-** | 18.2 | - |
| 2500.0 | 15.8 | **-** | 17.6 | **-** | 20.0 | - |
| 3000.0 | 18.0 | **-** | 19.8 | **-** | 22.2 | - |

Standard uncertainties are *k*_ψ_ = ±0.1 x 10^-5^ s^-1^.
